# Assessment of an ELISA method to support surveillance of bovine tuberculosis in Albania

**DOI:** 10.1186/s13620-016-0069-2

**Published:** 2016-08-20

**Authors:** Anita Koni, Arla Juma, Matteo Morini, Stefano Nardelli, Robert Connor, Xhelil Koleci

**Affiliations:** 1 Veterinary Public Health Department, Faculty of Veterinary Medicine, Agricultural University of Tirana, Kodër Kamëz, 1000 Tirana Albania; 2 Institute of Food Safety and Veterinary, Porcelan, Rr. “Aleksander Moisiu”, Nr.10, 1000 Tirana, Albania; 3 Istituto Zooprofilattico Sperimentale delle Venezie, Viale dell’Università 10, 35020 Legnaro, Padova Italy

**Keywords:** Bovine tuberculosis, *Mycobacterium bovis*, IDEXX ELISA test, Bovine TB seroprevalence, Bovine TB skin test, Albania

## Abstract

**Background:**

Bovine tuberculosis (bTB) is an important bacterial infectious disease in Albania of concern to animal and human health; its prevalence is poorly documented.

**Methods:**

In this longitudinal study, we tested by ELISA 2661 serum samples, from 154 herds, with the aim of establishing the suitability of this approach to screen the bovine population for bTB. In a follow-on survey of 87 animals in three villages, we assessed the usefulness of the *Mycobacterium bovis* IDEXX ELISA (IDEXX M. bovis Antibody (Ab) Test. IDEXX Europe B.V P.O. Box 1334, 2130 EK Hoofddorp, The Netherlands) assay by comparing IDEXX results with the results of the single intradermal cervical skin test. Skin tests were performed either after or at the time of collection of blood samples, and therefore cattle were not sensitized by tuberculin before serological testing.

**Results:**

The proportion of herds in which serologically positive cattle were found was 18.2 % (95 % CI, 1.9–25.8 %) and the prevalence of seropositive cattle was 1.4 % (95 % CI, 0.8–2.1 %). In the follow-up study, two of the 87 animals reacted positively to the skin test and two produced inconclusive reactions. No overlap was found between the four animals with positive IDEXX ELISA results and the four animals with non-negative skin test results.

**Conclusion:**

The lack of agreement between the results of the two tests may reflect different elements of the immune response (humoral and cell-mediated immunity). In future, cattle should be sensitized by the intradermal injection of tuberculin 14 days prior to the collection of blood samples, which would then be tested by the *Mycobacterium bovis* IDEXX ELISA Test in order to determine more accurately the prevalence of infection.

## Background

Bovine tuberculosis (bTB) is an important bacterial disease caused by *Mycobacterium bovis.* It occurs worldwide [1, 2] and affects a wide range of wild and domestic animals [2]. Bovine tuberculosis is a zoonotic disease: man can become infected via milk, aerosols, consumption of infected meat and accidental laboratory exposure [1, 3]. *M. bovis* is responsible for 5–10 % of all human tuberculosis cases [4] but the infection rate varies widely from country to country [1]. After exposure to infection, animals respond by activating both cellular and humoral immunity. *M. bovis* moves via the lymphatic system within cells to regional lymph nodes similar to other mycobacterial infections, and delayed hypersensitivity reactions typically commence between 30 and 50 days after the establishment of infection [2, 3, 5]. Tuberculosis is typically a chronic infection. Many infected animals in the herd remain undetected for a long time but they can shed the bacteria by aerosol, via milk, urine and faeces. Consequently, the infection spreads from chronically infected carrier animals to susceptible animals [3, 5]. Programmes to control bTB are based on an identification system for all animals, and the detection of infected individuals at post-mortem examination, especially during meat inspection. Intensive, systematic surveillance, commonly by means of skin tests, is usually linked to the slaughter of positive, reactor animals. Animal movement control and the cleansing and disinfection of contaminated environments [2, 5–9] are important adjuncts to effective control of bTB.

Despite the long history of attempted control and eradication, bTB remains an important disease and its prevalence - in global terms—is almost unchanged; in some countries its prevalence is increasing [1]. This picture is reported in developing countries and more developed countries, including those with highly organized and functional veterinary services that implement eradication programmes correctly [8, 9].

In Albania, the prevalence of bTB is poorly documented. After 1990, the cattle management system changed dramatically: the large, collective, state-owned farms were replaced by many thousands of privately owned small holdings, each with very few cattle. It is estimated that in 2012 there were 480,000 cattle in 375,217 holdings, 73 % with 1–4 animals, and only 5 % with more than 50 cattle. In epidemiological terms, these circumstances complicate the control of bTB. Conversely, the national cattle herd structure might reduce disease transmission since herds are small and are kept separated. Over the last 25 years, the bovine tuberculosis control programme has been based on a skin test, by use of a Purified Protein Derivative (PPD) bovine tuberculin produced by the national reference laboratory, the quality of which was not independently certified. On farms where reactor animals were found, no sanitary measures were implemented. During the last three years, no systematic control measures for bTB have been applied. Moreover, during recent years, there has reportedly been an increase in the prevalence of extra-pulmonary tuberculosis in humans from which *M. bovis* and *M. caprae* have been isolated (personal communication, Dr. Silva Tafaj)^1^, thus suggesting a bovine origin of the infection. Diagnostic tests that are available today have low sensitivity [2, 6–8, 10–12]. Active surveillance based on the skin test is expensive, and requires well-trained people along with field mobility, adequate supervision; specific, expensive equipment (syringes, needles, etc. not available in Albania.); certified tuberculin, etc.

Advances in the diagnosis of tuberculosis aim to introduce standardized, alternative tests with greater sensitivity and ease of use, and reduced costs, e.g. the *M. bovis* ELISA test (IDEXX®) [12, 13]. Available data suggest that the sensitivity of this ELISA test is increased by the previous stimulation of the immune system by the intradermal skin test [13], which is currently not done routinely in Albania. However, the higher age of Albanian cattle (frequently >10 years) in many holdings could give more time for them to develop a chronic humoral immune response detectable in the ELISA. For this reason, we conducted this survey to estimate the sero-prevalence of bovine tuberculosis using the IDEXX *M.bovis* ELISA test and to assess the suitability of this test for bTB surveillance in Albania, which could reduce costs of surveillance.

## Methods

This observational study was conducted according to the international guidelines stated in the strobe checklist (www.strobe-statement.org), this study was approved on 12 July 2013 by Department of Veterinary Public Health.

### National survey

We conducted a cross-sectional study in July 2013 for the presence of specific antibodies to *M.bovis* in bovine sera. Blood samples were collected from the jugular vein of cattle during a national bovine brucellosis survey and preserved in a serum bank, located at the Faculty of Veterinary Medicine, Tirana at −20 °C until the ELISA test was performed. Six ELISA kits were available and by means of lists of samples, we selected systematically every fifth sample from the serum bank, with the exception of samples from the Peshkopia district. We included and tested all available samples from the Peshkopia district since it is a known focus of bTB (personal communication, Prof. Pëllumb Muhedini).^2^ In total, 2661 sera were tested from 154 epidemiological units (communal herds), across 10 of the 12 regions and 20 of the 36 districts of Albania.

#### ELISA procedure

A tuberculosis antibody-screening test was used in accordance with the manufacturer’s instructions. The results of the ELISA tests were expressed as the value of the sample (S) divided by value of the positive control serum (P) supplied in the IDEXX ELISA kit, as determined by measurement of the optical density (OD_450_) with a “TECAN” ELISA plate reader.^3^ The reading obtained from each sample divided by the value of the positive control was used to calculate the S/P value for each sample.

### Follow on survey

Follow-up testing was conducted on cattle in three, purposively selected villages in the Peshkopia district: Pejke, Katund i Ri and Sohodoll. In each of these villages, all cattle were tested in parallel with the single intradermal cervical skin tuberculin test (SICST) and the *M. bovis* ELISA IDEXX Test. Jugular blood was collected for ELISA testing. The skin test was performed according to the procedure described in the EU directive 64/432/EEC by using specific devices, (i.e., 0,1 ml automatic syringe, caliper with 0,1 mm resolution—electric shears, and certified PPD bovine tuberculin^4^, (strain AN5 with a biological activity equal to 5000 IU). Blood samples were collected at the time of PPD injection and subsequently sera separated from these samples tested with the IDEXX ELISA kit. The skin test results were read 72 ± 2 h after the injection of tuberculin.

The criteria used to determine the status of animals tested by ELISA were as follows: an S/P value <0.3 was judged to be negative; an S/P value equal to or greater than 0.3 was considered positive. The criteria used to determine the status of animals tested by tuberculin test are shown in Table 2.

The data were analyzed in Microsoft Excel using the add-in Data Analysis toolPak (Descriptive Statistics).

## Results

Of the 2661 cattle tested, 1.4 % (95 % CI, 0.8–2.1 %) were ELISA positive. Of the 154 herds tested, 18.1 (95 % CI, 1.9–25.8 %) contained at least one ELISA positive animal. The ELISA results are presented in Table 1. Herd and animal prevalence did not vary significantly with district (chi-square for proportion of herds was 8.59 (*p* = 0.980), chi-square for proportion of cattle tested was 9.51 (*p* = 0.964)). Although no positive animals were detected in 7 of the 20 districts, neither herd or animal-level prevalence varied significantly between districts (*p* = 0.980 for herds, *p* = 0.964 for animals).

**Fig. 1:**
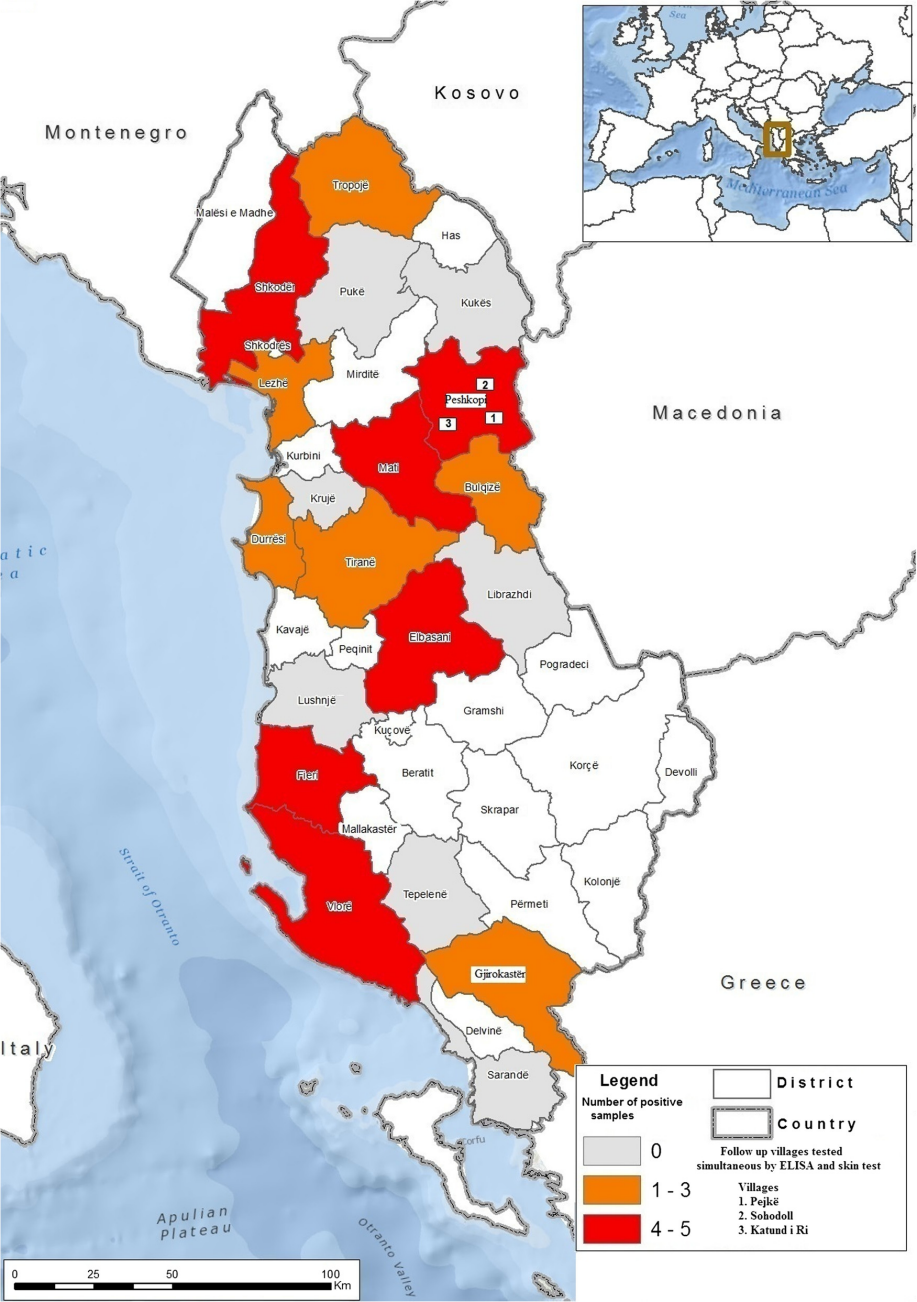
Map of districts sampled in Albania showing ELISA test results and follow on survey villages

In the follow on survey, two animals reacted positively to the skin test (Table 2); these were in the same farm in the village of Katundi i Ri (Fig. 1). Two animals produced inconclusive reactions (Table 2), one in Sohodoll and one in Katundi i Ri (Fig. 1). One of the two positive animals (a cow approximately 15 years old) had skin lesions and a fluctuating oedema, whereas the second reactor (a cow approximately 7 years old) showed only skin thickening at the injection site. The third animal present in the same farm (cow approximately 3 years old) was negative.

**Table 1:** ELISA test results of 2661 bovine sera blood samples in twenty districts of Albania

Districts	Number of tested herds	Number of positive herds	Percentage (%) of positive herds	Number of cattle tested	Positive samples number/percentage
Durrës	12	2	16.7	266	3/1.13
Krujë	2	0	0	10	0/0
Korçë	5	0	0	53	0/0
Shkodër	15	3	20	198	3/1.52
Fier	21	4	19	235	4/1.7
Lushnjë	6	2	33.3	123	2/1.62
Tepelenë	2	0	0	59	0/0
Tiranë	12	2	16.7	63	2/3.17
Peshkopi^a^	10	4	40	312	5/1.6
Sarandë	2	0	0	72	0/0
Vlorë	10	2	20	139	4/2.87
Elbasan	19	3	15.8	374	4/1.06
Lezhë	6	1	16.7	93	1/1.08
Pukë	4	0	0	44	0/0
Librazhd	1	0	0	13	0/0
Tropojë	6	1	16.7	38	1/2.63
Kukës	2	0	0	14	0/0
Bulqizë	6	1	16.7	148	1/0.67
Gjirokastër	5	1	20	126	2/1.59
Mat	8	2	25	281	4/1.42
Total	154	28	18.18	2661	36/1.35

*Legend:* Of the 2661 cattle tested, the percentage of positive animals was 1.4 % (95 % CI, 0.8–2.1 %). Of 154 herds tested, the percentage of positive was 18.2 % (95 % CI, 1.9–25.8 %)

^a^ In Peshkopia district, follow on tests were performed on 87 cattle in Katundi i Ri, Pejkë and Sohodoll villages

**Table 2:** Follow on skin test and ELISA test results on 87 cattle in three villages in the Peshkopia district of Albania

Criteria used for determining status of animals	Status of animals	Number of animals
Skin thickness difference ˂2 mm	Negative	83
Skin thickness difference 2–4 mm	Doubtful	2
Skin thickness difference >4 mm	Positive	2
ELISA test results	Negative	83
ELISA test results	Positive	4

*Legend:* Two animals produced positive skin reactions; four animals were positive in the ELISA test; no animal was positive in both tests

There was no overlap between positive or inconclusive skin tests and positive serological results. ELISA test results showed that four animals were positive (Table 2): one in Pejke, one in Sohodoll and two in Katundi i Ri (Fig. 1).

## Discussion

Many of the problems associated with bTB are due to the limitations of diagnostic tests [2, 5–7]. In general, sensitivity of official diagnostic tests for *M. bovis* infection is low, and is affected by several factors [2, 7]. Concurrent parasitic infections (e.g.*, Fasciola hepatica* and *M. bovis*) in cattle are known to reduce cell mediated immune responses to *M. bovis* [9]. However, this lack of sensitivity occurs even without concurrent *F. hepatica* coinfection [10]. These limitations complicate implementation of appropriate control measures.

In addition to skin tests, serological tests have been used to diagnose bTB in many countries. However, antibody detection tests, such as ELISA, are not capable of detecting sub-clinical infection [5, 13]. As the disease progresses and high numbers of *Mycobacterium bovis* bacteria accumulate in tissues, importantly, antibody levels increase in blood [5, 8, 11, 12]. At this stage, ELISA tests are useful for serological surveys of dairy herds. The sensitivity of ELISA tests is generally high in animals with clinical disease. A comparison of the skin tests and IDEXX *M. bovis* Test for diagnosis of bTB at the herd level, indicated that ELISA had a sensitivity of 63 % which is similar to the skin test [12]. The *Mycobacterium bovis* IDEXX ELISA Test has the advantage of being a more rapid test and it is less vulnerable to errors and biases on the farm.

In this survey, 1.4 % of the sera tested reacted positively for antibody to *Mycobacterium bovis*. The median specificity of the *M.bovis* IDEXX ELISA Test used has previously been estimated to be 98.2 % [12], which means that in a *M. bovis* negative population of 2661 cattle, up to 48 false positive results could have been expected. In our survey, 36 positive samples were found. Some of these positive results were from clusters of cattle. Households in Albania tend to keep cows for many lactations - a factor that could facilitate the detection of infected animals, since older animals could develop clinical disease. The lack of regular compulsory bTB testing in Albanian cattle meant that their immune systems would not have received the boosting effect of tuberculin.

When we compared the ELISA test with the classical skin test method some animals were positive on one or the other test, however no animals were found to be positive in both skin test and ELISA test. The ages of tested animals were much higher than in western European countries, i.e., the mean was 7.7 years: in Pejke 7.0 years, in Sohodoll 7.8 years, and in Katundi i Ri 8.8 years old. Under these conditions, the ELISA test should improve the possibility of finding chronically infected animals. We could not conclude, however, that the ELISA test is an effective test for monitoring bovine tuberculosis in the dairy cattle population. It would be useful to follow cattle that are positive in both tests through to slaughter to seek to detect histological changes that are characteristic of tuberculosis and to isolate *M. bovis* from lesions and tissues. Since the two tests we used measured different immune responses, it may be useful to use both testing strategies, ideally using the *Mycobacterium bovis* IDEXX ELISA Test 2 weeks after the skin test [5], to screen the bovine population for the prevalence of bovine tuberculosis*.* With the serological boost resulting from tuberculin, the cut-off could be increased, probably resulting in increased specificity.

### Limitations of this survey

The following limitations were identified during this survey:

- Lack of appropriate identification and registration of cattle in Albania (57.5 % of cattle in follow on survey were not properly identified/ear tagged).
- Lack of animal movement control: the cow that had the highest ELISA S/P value (6,3), had been sold and was not available for the skin test or for further follow up by ELISA testing.
- Lack of scientific evidence on the current bTB in Albania, especially on a national scale.
- Lack of resources for further follow up of positive animals detected in slaughterhouses by herd skin or ELISA tests.

## Conclusions

The number of positive animals detected in ELISA test was low. This could reflect the poor sensitivity of the ELISA test on sera from animals that were not previously sensitized by tuberculin skin test. To produce more accurate results, it is suggested that in future all cattle in affected holdings (herds) should be sensitized by means of the tuberculin skin test preferably 14 days prior to the collection of blood samples, which would then be tested by the *M. bovis* ELISA IDEXX Test. An affordable and practical approach is needed to control bTB in Albania. Consequently, we recommend that if a suspicious lesion is found at a slaughterhouse, the herd of origin of the animal should be traced, and a skin test and *M.bovis* IDEXX ELISA Test should be performed on the herd. Tuberculin skin test should be performed by adequately trained veterinarians. Movement control is an essential sanitary measure.

## Abbreviations

Ab, antibody; bTB, bovine Tuberculosis; CI, confidence interval; ELISA, enzyme-linked immunosorbent assay; EU, european union; *F.Hepatica*, *Fasciola Hepatica;* IU, international unit; *M.Bovis, Mycobacterium Bovis;* OD, optical density; P, positive control serum; PAZA, protection against zoonotic diseases (Albania); Phd, philosophy doctor; PPD, purified protein derivate; S, sample; SICST, single intradermal cervical skin tuberculin test

## Footnotes

1 Human Pulmonary University Hospital, Head of *Mycobacterium tuberculosis* laboratory, Tirana

2 Former Head of Bio-products production, Veterinary Research Institute, Tirana, Albania

3 Manufacturer, Tecan Austria GmbH (“Tecan”) 5082 Grödig, Austria

4 PPD bovine tuberculin produced by the “Istituto Zooprofilattico Sperimentale dell’Umbria e delle Marche” (Perugia, Italy) and officially approved by the Italian National Reference Laboratory
